# Design of a tobacco exon array with application to investigate the differential cadmium accumulation property in two tobacco varieties

**DOI:** 10.1186/1471-2164-13-674

**Published:** 2012-11-28

**Authors:** Florian Martin, Lucien Bovet, Audrey Cordier, Mario Stanke, Irfan Gunduz, Manuel C Peitsch, Nikolai V Ivanov

**Affiliations:** 1 Philip Morris International R&D, Philip Morris Products SA, Neuchatel, 2000, Switzerland; 2 Institut für Mathematik und Informatik, Greifswald, D-17487, Germany; 3 Philip Morris International Operations, Neuchatel, 2000, Switzerland

**Keywords:** Tobacco plant, Nicotiana tabacum, Exon array, Gene expression, Cadmium accumulation

## Abstract

**Background:**

For decades the tobacco plant has served as a model organism in plant biology to answer fundamental biological questions in the areas of plant development, physiology, and genetics. Due to the lack of sufficient coverage of genomic sequences, however, none of the expressed sequence tag (EST)-based chips developed to date cover gene expression from the whole genome. The availability of Tobacco Genome Initiative (TGI) sequences provides a useful resource to build a whole genome exon array, even if the assembled sequences are highly fragmented. Here, the design of a Tobacco Exon Array is reported and an application to improve the understanding of genes regulated by cadmium (Cd) in tobacco is described.

**Results:**

From the analysis and annotation of the 1,271,256 *Nicotiana tabacum* fasta and quality files from methyl filtered genomic survey sequences (GSS) obtained from the TGI and ~56,000 ESTs available in public databases, an exon array with 272,342 probesets was designed (four probes per exon) and tested on two selected tobacco varieties.

Two tobacco varieties out of 45 accumulating low and high cadmium in leaf were identified based on the GGE biplot analysis, which is analysis of the genotype main effect (G) plus analysis of the genotype by environment interaction (GE) of eight field trials (four fields over two years) showing reproducibility across the trials. The selected varieties were grown under greenhouse conditions in two different soils and subjected to exon array analyses using root and leaf tissues to understand the genetic make-up of the Cd accumulation.

**Conclusions:**

An Affymetrix Exon Array was developed to cover a large (~90%) proportion of the tobacco gene space. The Tobacco Exon Array will be available for research use through Affymetrix array catalogue. As a proof of the exon array usability, we have demonstrated that the Tobacco Exon Array is a valuable tool for studying Cd accumulation in tobacco leaves. Data from field and greenhouse experiments supported by gene expression studies strongly suggested that the difference in leaf Cd accumulation between the two specific tobacco cultivars is dependent solely on genetic factors and genetic variability rather than on the environment.

## Background

Tobacco (*Nicotiana tabacum* L.) is a species in the large family of the Solanaceae and is important as an agronomic crop, since more than six million tons of tobacco are produced per year throughout the world. For decades the tobacco plant has served as a model organism in plant biology and has helped to answer fundamental biological questions in the areas of plant development, physiology, and genetics. It has made its scientific reputation due to the ease of gene transformation and genetic manipulation. Tobacco is an allopolyploid species (2*n*=4x=48) and shares its basic chromosome number of x = 12 with many other Solanaceae species, such as tomato, potato, pepper, and eggplant. It is most likely the result of a tetraploidization event [1,2] involving *Nicotiana sylvestris* (S-genome) and a species closely related to modern day *Nicotiana tomentosiformis* (T-genome). Considering both genomes together, tobacco is at the high end of genome sizes (4.5 Gbp) in the Solanaceae [3] and contains a large proportion of repetitive sequences [4,5].

The North Carolina State University (NCSU) Tobacco Genome Initiative (TGI) was started in 2002 in cooperation with Philip Morris USA to gather genetic information on *N. tabacum* by means of sequencing gene-rich regions of genomic DNA and cDNA libraries of Hicks Broadleaf variety. The TGI website (http://www.pngg.org/tgi/) contains related project information and links for data download. TGI has been leveraged to build a dense tobacco genetic map [6] and microsatellite marker kits for variety identification [7]. Many phenotypes of importance in tobacco, such as heavy metal accumulation, nutrient deficiency, and yield, are thought to be transcriptionally controlled. Therefore, microarray technology is a suitable tool to study genetic variation and environmental effects with the objective to improve varieties of crops.

Expressed sequence tag (EST)-based tobacco microarrays have been used successfully in tobacco plant research [8,9]. Cui *et al.* compared the gene expression of 2,831 selected tobacco genes between the trichomes and the leaves with removed trichomes. Trichomes predominantly expressed genes involved in the second metabolic processes, defence responses, and metabolism regulation [8]. A Tobacco Expression Atlas (TobEA) [9] was constructed through systematic measurements of gene expression across different tobacco samples. To achieve this, a custom-built Affymetrix GeneChip™ was designed from the cDNA sequences originating from multiple tissues (seeds, roots, leaves, flowers, etc.) of several tobacco varieties. However, due to the lack of sufficient coverage of genomic sequences before the TGI data were released, none of these arrays were intended to cover genome-wide gene expression.

There is evidence that in many plant genomes, including tobacco, the repetitive regions are heavily methylated and the gene regions are undermethylated [10]. Consequently, a methyl filtration approach was used in the TGI to reduce the complexity of tobacco genome sequencing and at the same time to cover a large portion of the coding regions in the genome.

The challenge was to design a whole genome functional chip that can be used to provide a more consistent and comprehensive picture than an EST-based microarray. Because the assembly of unmethylated tobacco genome sequences yields highly fragmented contigs covering a large set of functional genes representing gene-rich portion (~13%) of the genome, this exon array is a feasible alternative to a full genome gene array. In this study we present the application of the Tobacco Exon Array to measure differential gene expression with the objective to improve our understanding of cadmium (Cd) accumulation by the tobacco plant.

Cd accumulation in crop plants such as tobacco can lead to human exposure to this carcinogenic metal. Therefore, there is a considerable interest to find strategies to produce plants with low Cd content. Tobacco tends to sequester higher concentrations of Cd in the leaves than in the roots. The Cd concentration in tobacco leaves usually ranges from 0.1 to 5 μg/g dry weight; the Cd concentration in lower leaves is usually more elevated compared to higher and mid/upper-stalk position leaves [11]. In addition, large differences in leaf Cd content exist among tobacco varieties and between growing regions [12], which suggests the predominant role of both genetic background and environmental conditions.

In this study, to better understand the genotype-environment interactions, eight field trials over a period of two crop years were performed to identify two variety candidates that differentially accumulate Cd in leaves, independently of the environment. The Tobacco Exon Array was then used to unravel the difference in transcription between those two varieties.

## Results

### Design of tobacco exon array

Due to the large size and complexity of the tobacco genome, a methyl filtration approach [10,13,14] was used within the TGI to enrich the genomic clone library with gene-rich genomic DNA sequences [15]. At the time of the study (June 2007), 1,271,256 *N. tabacum* fasta and quality files from methyl filtered genomic survey sequences (GSS) were obtained from the TGI. The cleaning procedure with seqclean removed 13,010 reads matching known vectors, including the ones used by TGI, and 146 reads originating from *E. coli* contamination. Further quality control scripts removed 23,883 mitochondrial and plastid reads, 245 short reads, and 257 reads containing undetermined bases, resulting in 1,233,715 reads. Simple repeats were identified and masked in 114,897 (9.0%) reads. Complex repeats were identified and masked in 156,899 (12.7%) reads. In the absence of tobacco plant-specific repeats, repeat libraries containing retrotransposons and DNA transposons of *Brassica*, maize, rice, barley, wheat, and *Solanum* family from mips-REdat [16] and SOL Genomics Network [17] were used. The final set contained 1,172,176 reads adjusted for cleaning and trimming. Due to lack of sufficient genome coverage (<1X) of the data (even over the euchromatin part of the genome estimated to be ~500 Mbp based on the tomato genome), the tobacco genome could only be partially assembled. Celera assembler produced 387,927 singletons and 183,198 contigs with the N_50_ = 990 (contigs only) and 4.28 reads per contig, resulting in ~0.13X coverage of the tobacco genome.

**Figure 1:**
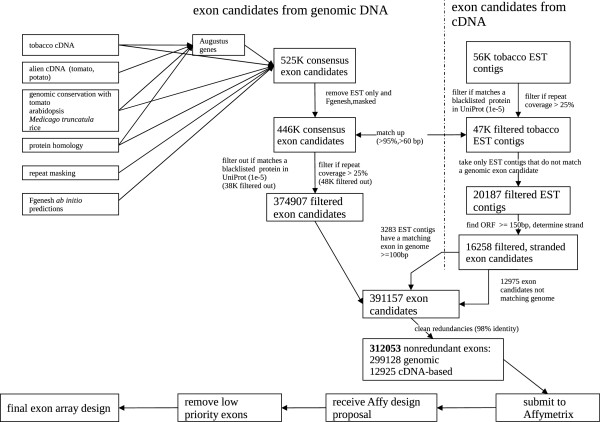
**Flow diagram of the exon identification for the Affymetrix chip design.** Candidate exons were extracted from genomic DNA and cDNA with the successive filtering steps.

Exon candidates for the Affymetrix GeneChip Tobacco Exon 1.0 ST Array were identified from two main sources: exons predicted with AUGUSTUS and FGENESH within the tobacco genomic assembly and exons from the alignment of tobacco ESTs to the assembly (Figure 1).

**Figure 2:**
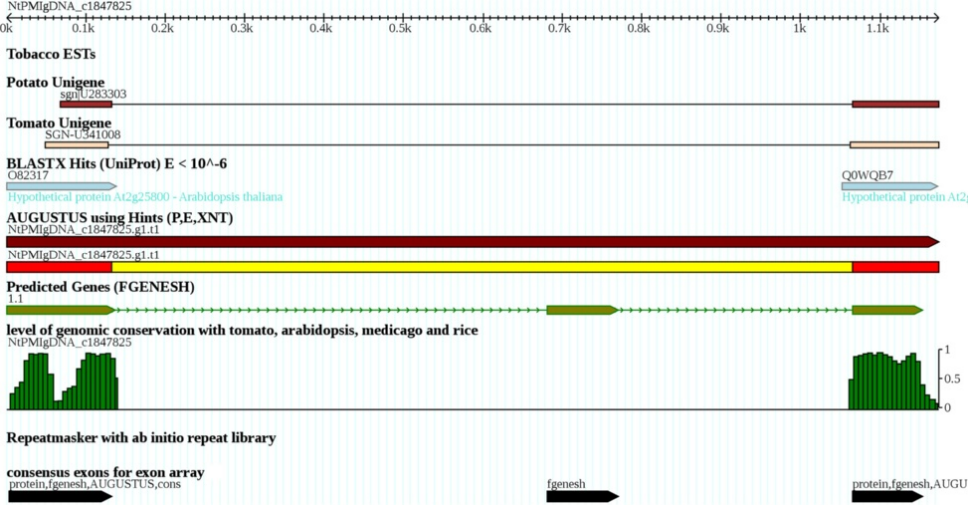
**Example of evidence used in gene prediction.** As typical for this assembly, the genomic contig contains only part of a gene; shown are two exons that are truncated at the contig ends.

Because the genome assembly was highly fragmented and the majority of genes were only partially contained in a genomic sequence (contig), we further adjusted AUGUSTUS to predict the partial genes. The contig start and end were allowed to truncate a gene structure of AUGUSTUS at any location in a coding exon, a UTR exon, or an intron (see Figure 2). This new version, which could predict arbitrarily truncated gene structures, was tested on known genes whose contigs were artificially randomly truncated using the same size distribution as the genome assembly and which achieved an exon-level sensitivity of 54% and specificity of 62% in this test. For comparison, on the same test set exon-level sensitivity and specificity of FGENESH were 31% and 35%, respectively.

As described in the Methods section, the evidence from various sources for exons (*ab initio* predictions, transcript sequences, homology within related species, conservation at genome level) and against exons (repeats) was integrated towards a set of ~525,000 consensus exonic regions and passed as hints to AUGUSTUS to predict exact exon boundaries. Each exon was assigned a score based on the number of supporting evidence sources. The exons were further filtered to remove those with a lower degree of evidence, resulting in 374,907 exon candidates.

At the same time, about 56,000 contigs of ESTs were used to identify transcript fragments not already included in the genomic exonic regions. In order to determine the strand, an open reading frame (ORF) of at least 150 bp was required. EST contigs that appeared to be redundant with the exonic regions from the genome were removed. The resulting 312,053 exonic regions were scored by supporting evidence (0–7) so they could later be prioritized after further filtering by Affymetrix and after determining the exact size of the exon array. Finally, 272,342 exons were included to make 1,089,368 probes (four probes per exon) selected by Affymetrix.

### Field trial results

**Figure 3:**
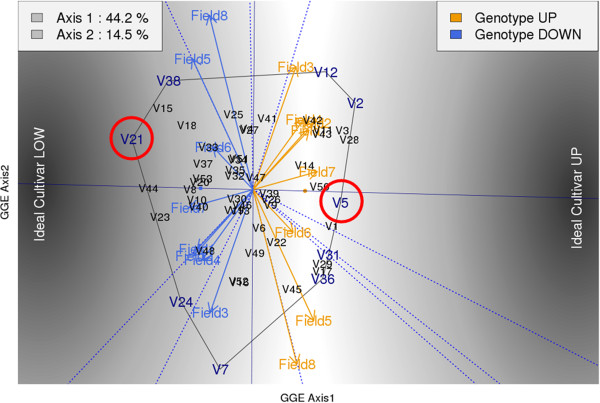
**GGE axes 1 and 2 show the mapping of all varieties involved in the eight field trials (explaining ~60% of the inertia).** The ideal cultivars are those with constant lowest and highest Cd values, respectively. The line relating those two represents the cultivars in which the Cd is expected to be constant across environments. This leads to the choice of the susceptible varieties (cultivars): V21 and V5.

The purpose of the analysis was to understand the relationship between cultivar performance regarding Cd uptake and the environment (= Field x Year). The ideal variety would be a variety with low Cd content in leaves that is stable across the environment. As expected, we observed significant Genotype (G), Environment (E), and Genotype by Environment interaction (GE) effects. GGE biplot analysis aims to explain the G+GxE as g_1_e_2_+g_2_e_2_, where g_i_ is the genotype eigenvectors and e_i_ the environment vectors (Figure 3). The data show that variety 21 (V21) and variety 5 (V5) accumulated low and high Cd across different environments, thereby suggesting that the difference in shoot Cd sequestration was due mainly to genotypic differences. In fact, V21 is a flue-cured tobacco and V5 is a burley tobacco, and the data are in accordance with the literature showing that burley varieties generally accumulate more Cd than flue-cured varieties [12]. We also compared two varieties under the same environmental conditions and field practices, which was not done in previous studies [11,12]. In Figure 3, it can be seen that variety V44 is also a low Cd accumulator, however, as it is a more exotic variety less cultivated worldwide than V21, V44 was not selected.

Table 1 shows the Cd values for both V21 and V5. Under the eight field conditions (two countries over two years under burley and flue-cured fertilization regimes), V21 accumulated on average 32% less Cd than V5.

**Table 1:** Cadmium concentrations for V21 (flue-cured) and V5 (burley) grown in field trials

**Field**	**V21 (mg/kg)**	**V5 (mg/kg)**	**P-value**	**P-value 2 (Corrected data)**	**Relative decrease**
1	2.6 ([2.02,3.18])	3.95 ([2.96,4.94])	0.02	0.00	34.20
2	1.82 ([1.35,2.3])	2.26 ([1.7,2.81])	0.16	0.15	19.20
3	0.81 ([0.54,1.08])	1.23 ([0.95,1.5])	0.02	0.00	33.90
4	0.84 ([0.56,1.11])	1.09 ([0.85,1.33])	0.11	0.02	23.40
5	1.2 ([0.99,1.4])	2.58 ([2.28,2.89])	0.00	0.00	53.70
6	1.33 ([1.09,1.58])	2.53 ([1.58,3.48])	0.02	0.01	47.40
7	2.5 ([2.17,2.83])	2.9 ([2.18,3.62])	0.24	0.00	13.80
8	1.78 ([1.4,2.17])	2.87 ([2.49,3.25])	0.00	0.00	37.80

Values in brackets are 95% confidence intervals; P-value 2 is the P-value for the *t*-test on spatially corrected data indicating the real genotype effect. Relative decrease is calculated as (V5-V21)/V5*100.

To perform transcriptomic analysis, V21 and V5 were also grown under controlled conditions in the greenhouse to identify putative gene candidates involved in Cd accumulation, since low variability were found in the field.

### Greenhouse experiment with V21 and V5

The two varieties were grown under greenhouse conditions in two different soils containing different Cd content (0.085 ppm for soil 1 and 0.12 ppm for soil 2). The levels of Cd in the pooled leaf samples of each variety were similar to those was observed under field conditions, thus confirming the choice made based on the GGE analysis and Cd susceptibility. In addition, the relative decrease between the two varieties (Table 2) is in the same range of the average differences observed in the field trials (Table 1).

**Table 2:** Cadmium concentrations in leaf and leaf weight for V21 (flue-cured) and V5 (burley) grown in the greenhouse n two different soils

**Cd (mg/kg)**	**V21**	**V5**	**P-value**	**Relative decrease**
Soil 1	0.43 ([0.37,0.49])	0.72 ([0.6,0.84])	0.00	40.5%
Soil 2	0.52 ([0.47,0.56])	0.82 ([0.73,0.92])	0.00	37.4%
**Leaf Weight (g)**				
Soil 1	48.3 ([42.8,53.79])	52.59 ([49.13,56.06])	0.13	8.2%
Soil 2	39.53 ([36.84,42.21])	37.44 ([32.81,42.07])	0.35	−5.6%

Values in brackets are 95% confidence intervals.

### Quality control (QC) for the tobacco exon array

**Figure 4:**
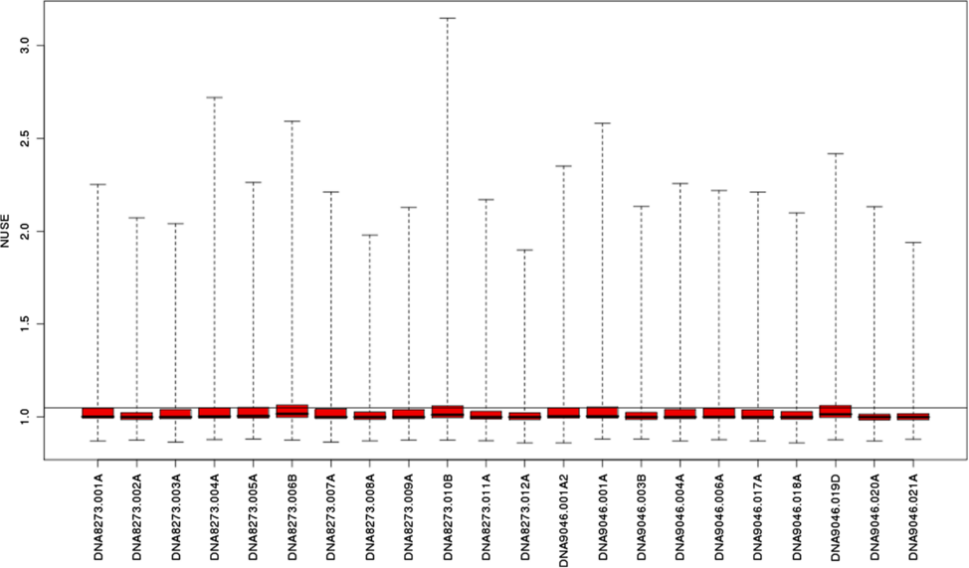
**Normalized Unscaled Standard Error (NUSE) plot.** Any array with a median value above 1.05 is considered an outlier.

**Figure 5:**
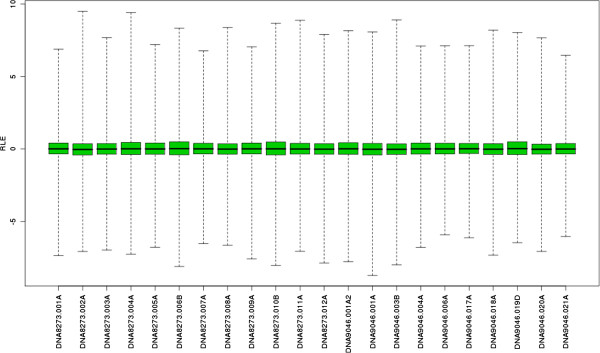
**Relative Log Expression (RLE) plot.** Boxplots are expected to have a small interquartile range (IQR), otherwise the arrays are considered as outliers.

The greenhouse samples were subjected to microarray analysis. All quality control (QC) metrics indicate that the chip design, together with the hybridization protocol used, is overall very satisfactory (only a few low quality chips were found). For instance, in the experiment described here, only one of the 22 chips used was rejected (see in particular Figures 4 and 5).

**Figure 6:**
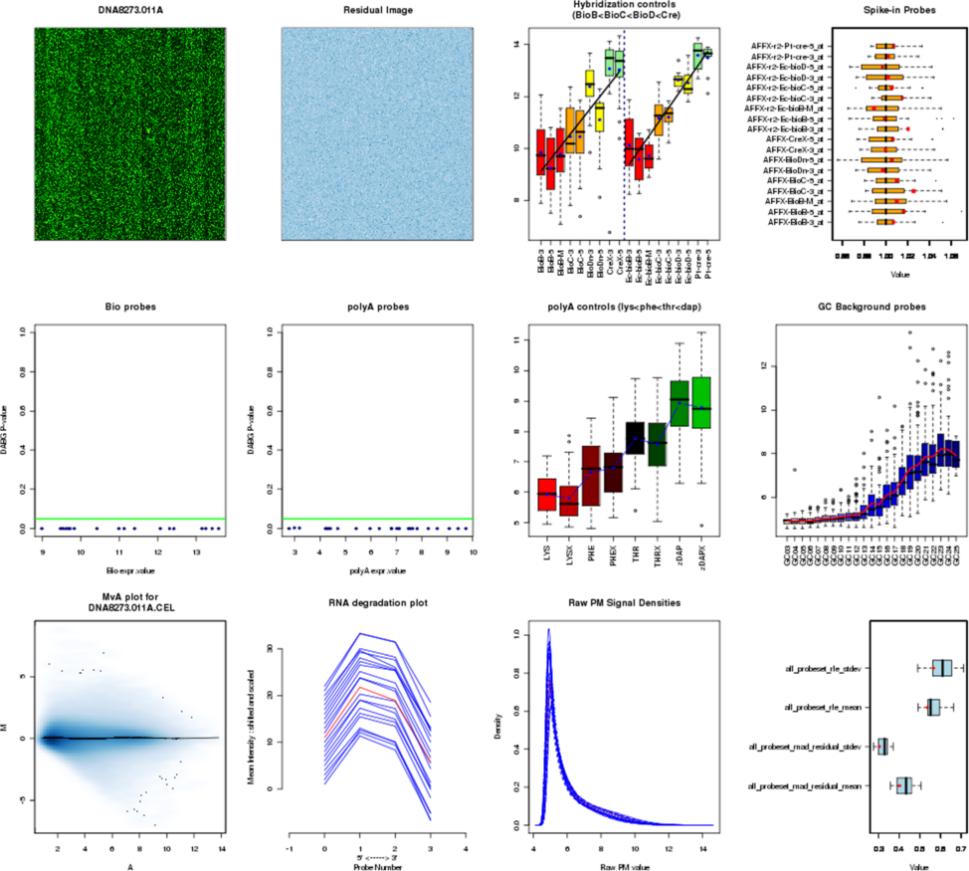
**A typical individual chip quality control showing its scanned image, pseudo-image, control probes study, random GC probes, MvA plot, RNA degradation plot, raw signal density, and residual study.** (See references in the text.).

One specificity of this chip design is the unusual shape of the RNA degradation plot (see Figure 6). Another feature is the Detection Above BackGround (DABG) P-value [Affymetrix White Paper, Exon Array Background Correction http://media.affymetrix.com/support/technical/whitepapers/exon_background_correction_whitepaper.pdf] used to filter out some probesets prior to analysis. Based on about 200 chip hybridizations, the proportion of probesets showing at least one DABG P-value lower than 0.05 was 84%. In the Cd experiment the percentage was still high (70.7%).

### Gene expression analysis (set of tools to analyze exon array)

Once the chips meet the quality standard of the QC metrics, the probesets with high DABG P-values were filtered out (threshold was set to 0.1). The experimental design led to the definition of a linear model (see Methods) that was fitted for each probeset using LIMMA [18] R package. For each coefficient of the linear model, P-values were adjusted by Benjamini-Hochberg false discovery rate (fdr) correction [19]. Finally the probesets of interest were selected based on coefficients and fdr P-value thresholds (0.05). Because many of probesets had high coefficients, we also put a threshold on the coefficient values (3 on the log2-scale, corresponding to an 8-fold change) to be able to interpret the results.

**Figure 7:**
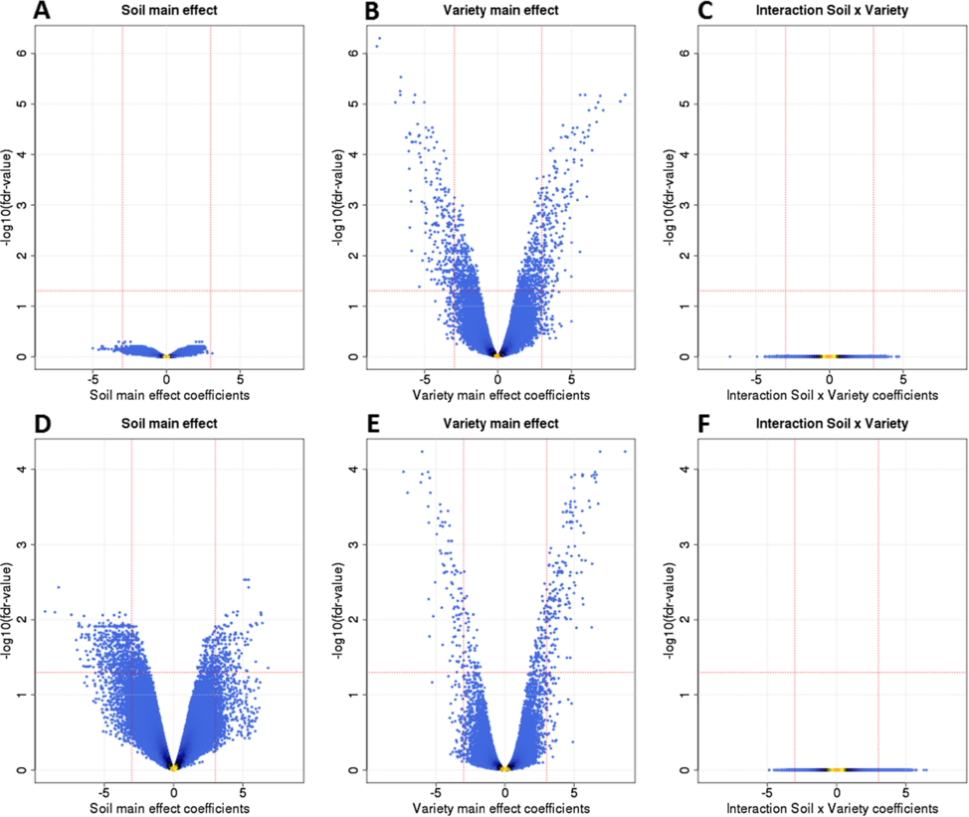
**Volcano plots.** Log2 effect vs –log10(fdr values); **A**) Soil main effect for leaf samples; **B**) Variety main effect for leaf samples; **C**) Soil x Variety interaction effect for leaf samples; **D**) Soil main effect for root samples; **E**) Variety main effect for root samples; **F**) Soil x Variety interaction effect for root samples.

For the variety main effect, 444 probesets were identified as differentially expressed exons in leaves and 265 in roots (see Figure 7, middle panels); 147 exons were common to both. Soil effect was observed in roots (672 exons differentially expressed), but not in leaves (Figure 7, compare lower and upper left panels). No Soil x Variety interactions were observed (Figure 7, right panels).

The highest differentially expressed exons (absolute coefficient values above 5) are shown in the Additional file 1: Tables S1 and S2. For these transcripts, the number of differentially expressed exons in leaf is actually well-distributed between V5 and V21; 29 exons are highly expressed in V21 and 32 in V5. This suggests that the two varieties have each a specific set of expressed exons in leaf contributing to the known physiological differences observed between flue-cured tobacco (V21) and burley tobacco (V5), i.e., color of the stems and leaf thickness. However, such a gene expression pattern may contribute to the differential Cd sequestration profiles within the leaves of the two varieties as shown in Tables 1 and 2. Interestingly, the only exon-related gene that could play a specific role in Cd accumulation corresponds to a MATE transporter as identified by the probe NtPMIa1g52548e2_s_st (5’-CAGTACGACAATTCTAGGGTGGGTATTCATGATTTCTCTTGGCTTCAATGCAGCAGCAAG-3’). MATE transporters have already been reported to transport metals such as aluminium in barley and Arabidopsis [20,21], and to be regulated upon Cd stress [22] or involved in Cd detoxification and tolerance [23]. In order to support the biological interpretation of the experiment, gene set enrichment analysis was performed for each contrast (e.g., Variety effect and Soil effect) using biological function-associated gene sets as a priori knowledge (see Methods). Thirty-six significant gene sets (fdr<0.01) were identified for the variety effect in leaves (see Additional file 1: Table S3). Among them, ABC transporters, known to be involved in Cd transport in plants [24-26] may play a role in the differential Cd accumulation and/or sequestration in leaves of both species. Besides ABC transporters, six other gene sets are related to transcription factors and seven gene sets to other type of transporters, both may contribute to leaf Cd sequestration. Other generic gene sets linked to light photosynthesis reactions and sugar pathways are certainly more specific markers for burley and flue-cured tobacco.

In roots, the difference between V5 and V21 was more apparent: 31 exons were highly expressed in V21, but only 14 exons in V5. In this set of genes, no probeset annotations were found to be directly linked to Cd uptake, sequestration, or translocation to the shoot. In V21, more genes likely contributing to higher active root Cd sequestration were activated than in V5. In addition, only 5 significant gene sets (fdr<0.01) were found in roots (root variety effect, see Additional file 1: Table S3), suggesting that only a few specific root functions are different between V5 and V21. In conclusion, microarray analysis confirmed that the Cd accumulation trait is linked to a constitutive differential transcriptional gene expression of multiple genes explained by general genetic variability between V5 and V21 and not due to soil properties.

The analysis of the set of root genes responding to the soil effect shows that 132 gene sets (see Additional file 1: Table S3) play a role by activating or deactivating several root functions, depending on the soil composition. Among these gene sets, the major gene families involved in Cd transport, sequestration, root-to-shoot translocation, and Cd cell responses are represented, namely “ABC transporters” [27,28], “metal”, “cell wall general”, “cell wall lignification” [29], and “callus formation” [30], “enzyme ascorbate glutathione cycle” [31], “stress_GST”, “formation of GST-complexes” [32], ”Ca^2+^ cation antiporter” [33], “Multidrug Resistance exporter” (vacuolar Cd sequestration via MRP transporter) [34]; “Pleiotropic Drug Resistance protein” [26,35], and “P-type ATPase” (Cd root-to-shoot translocation) [36], and “Cd sequestration” [37]. According to the literature, these sets of genes cover the main pathway driving Cd from root uptake to leaf storage. Interestingly, although the transcription of these gene sets is affected by the soil environment, no major differences in Cd were found in the leaf between field trials and greenhouse experiments (compare relative decrease between Table 1 and Table 2). This suggests that plants adapt to the soil environmental conditions to maintain fitness and optimal growth conditions. The resulting effect is visualized by Cd content which is not affected or only slightly affected by the environmental conditions.

Altogether, these data indicate that only a few Cd-induced genes are involved in the differential accumulation of Cd between the flue-cured variety V21 and the Burley variety V5. Thus, a global genetic variability between these two tobacco species likely exists, including differential constitutive expression of certain genes. Nevertheless, we cannot exclude the possibility that Cd variation may also result from post-translational regulation involving specific proteins in one or the other variety.

### Semi-quantitative RT-PCR validation

**Figure 8:**
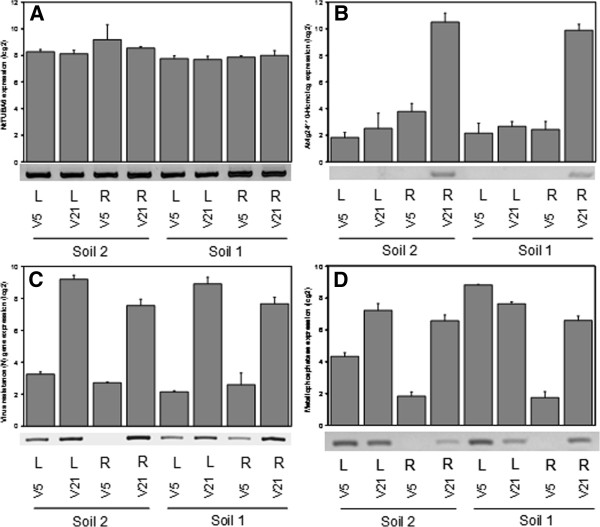
**RT-PCR validation of four selected genes.** The barcharts show the probeset expression values as measured by the Exon Array; the corresponding RT-PCR data are shown below. **A**) NtTUBA6 house-keeping gene. **B**) At4g24110-homolog gene **C**) Virus resistance (N) gene **D**) Metallophosphatase gene.

To validate the microarray data analysis, we performed semi-quantitative RT-PCR using primers designed in the exon sequence area matching the four probes (Figure 8). We found that semi-quantitative RT-PCR validated more than 90% of the hybridized exon probes examined, indicating that the expression data generated by this exon array are accurate. For this particular experiment, we designed specific primers for four selected genes (see Methods), including one house-keeping gene encoding tubulin A6 (NtTUBA6) and three selected genes coding for a homologue of At4g24110, a metallophosphatase, and a virus resistance (N) gene. As the Cd accumulation difference is due mainly to constitutive genetic variation, and not to the soil effect (see section above), we randomly chose three genes that are differentially expressed between V5 and V21. Semi-quantitative RT-PCR data were in accordance with the exon array results, thereby indicating that the Tobacco Exon Array results can be independently confirmed. *NtTUBA6* is a suitable transcript that can be used as a house-keeping gene for semi-quantitative RT-PCR. The tobacco homologue of At4g24110 is exclusively expressed in the roots of V21 and not in V5. This gene codes for a plant-specific HUP (Hypoxia-responsive Unknown Protein [38,39]) that influences low-oxygen stress tolerance. As the transcription difference is also verified by RT-PCR, it is therefore possible that the sensitivity to anoxic conditions is different between V5 and V21. Metallophosphatases are acid phosphatases using metal ligands and having substrate specificity towards phytate, diphosphate nucleosides, and inorganic pyrophosphate [40]. This tobacco metallophosphatase is not expressed in the V5 roots. Although not detected as a significant interaction (high fdr, >0.05), this gene was slightly down-regulated in V5 grown in soil 2. The virus resistance (N) gene is more expressed in V21 than in V5, and may be differently involved in protecting tobacco against viral infection, thereby possibly providing elevated resistance to V21 than to V5.

## Discussion

While the whole tobacco genome sequence is still not completed, the TGI has provided the scientific community with valuable sequence information that has created opportunities to develop plant biology tools, such as the Tobacco Exon Array. The Tobacco Exon Array was built to include a large set of experimentally confirmed and predicted genes within the tobacco gene space reaching an estimated coverage of more than 90%. Many applications of exon arrays have been developed for other plant species [41]. Our experience with the Tobacco Exon Array demonstrated that it is a powerful tool, as shown in the present study on Cd accumulation.

Across all internal experiments, only a few chips were discarded after examination of the QC metrics. The DABG calls were used to eliminate probesets prior to statistical analysis and few expression data were contradicted by RT-PCR validation. It should be stressed, however, that due to the fragmented nature of the draft genome of *N. tabacum*, the Tobacco Exon Array was analyzed as a gene chip disregarding alternative splicing events, even though this type of analysis can be performed for well annotated multi-exon genes [42-44].

The experimental goal was to find suitable tobacco varieties exhibiting reduced Cd translocation from root to shoot and ultimately to discover genes involved in leaf Cd accumulation. The field experiments allowed us to highlight two tobacco varieties differentially accumulating Cd that were stable across the environments (avoiding the GxE effect), thereby enabling greenhouse experiments to identify gene transcripts involved in Cd accumulation using Tobacco Exon Array.

**Figure 9:**
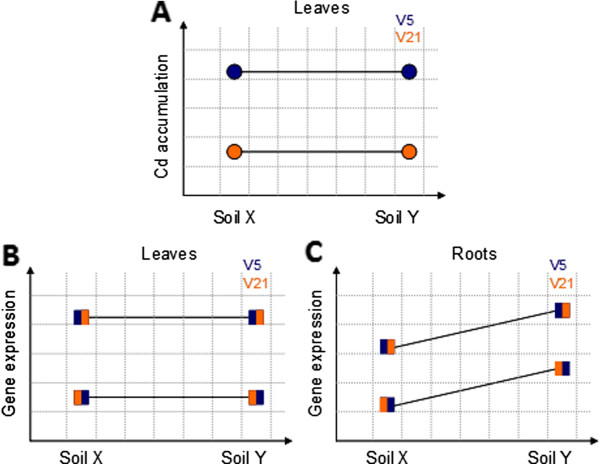
**Schematic summary of Cd accumulation experiments.A**) Cd accumulation in leaves is due solely to the genetic makeup of the varieties and is not linked to any soil composition; **B**) Gene expression in leaves correlates with the pattern described in A (either gene expression is higher ([left part of square box] or lower [right part of square box] in V5 than in V21); **C**) Interaction effects in the roots show a dependence on both genetic makeup and soil composition.

Figure 9 displays a scheme summarizing the observed effects on Cd content and soil effects on gene expression in leaves and roots and in between varieties. We observed that modification of the soil composition induced a lot of transcriptional fluctuations, but those effects mostly stayed localized in the root organs without any major changes at the leaf level. Consequently, Cd content in leaves remained similar in both soils, thereby indicating that *N. tabacum* has mechanisms to adapt to the soil environment conditions maintaining physiological steady-state at the leaf level. This is highlighted by a global Cd phenotype possibly due to the tuning of the transpiration rate, root-to-shoot ion fluxes or other root metabolic activities. For instance, in *Phytolacca americana* transpiration plays an important role in Cd accumulation in shoots [45] and Cd accumulation in shoots is driven by root-to-shoot translocation via the xylem in rice [46] and other crops [47]. The major genes known to be involved in the root-to-shoot translocation belong to the family of HMA (Heavy Metal ATPase), and particularly HMA2 and HMA4 in Brassicacea [36,48]. Interestingly, HMA2 and HMA4 related exons did not show major expression difference between V5 and V21, thus suggesting that they do not play a major role in the differential Cd accumulation. Cd sequestration and transport are certainly the key steps conducting to Cd phenotype differences within the leaves and are mainly explained in this particular case by the genotype constitutive difference between the two tobacco accessions. Such positive correlations established between genetic variation and Cd accumulation in roots of poplar trees [49]. Such type of gene expression adaptations to maintain key metabolic and steady-state activities have been described in the literature for plant respiration [50] and major seed storage proteins [51]. However, we cannot exclude an interaction effect between soil concentrations (low/high) of cadmium and the genetic background.

## Conclusions

An Affymetrix Tobacco Exon Array was developed based on the current genome and EST sequence data from the TGI to cover a large proportion of the tobacco gene space. The advantages of the exon array design include (i) representation of the genes not yet found in the currently available EST libraries, (ii) ability to investigate alternative splicing in the tobacco plant, and (iii) equal probe coverage of each exon of the gene. The Tobacco Exon Array will be available for research use through Affymetrix array catalogue. The experimental data described in this work have been released to Gene Expression Omnibus (GEO) under accession number GSE42319 (http://www.ncbi.nlm.nih.gov/geo/query/acc.cgi?acc=GSE42319) and will also be publicly available through GeneVestigator (https://www.genevestigator.com/gv/plant.jsp) or SGN (http://solgenomics.net) web sites.

As a proof of the exon array usability, we have demonstrated in this study that the Tobacco Exon Array is a valuable tool for studying stresses in tobacco, in particular Cd accumulation in leaves. Data from field and greenhouse experiments supported by gene expression studies strongly suggest that the difference in leaf Cd accumulation between the two specific tobacco cultivars is dependent solely on genetic factors and genetic variability.

## Methods

### Tobacco genomic read assembly and gene prediction

Fasta and quality files generated using phred (Phil Green, http://www.phrap.org) from *N. tabacum* methyl filtered GSS reads were provided by North Carolina State University as a part of the TGI [15]. A cleaning script seqclean (http://sourceforge.net/projects/seqclean/) with “-A -L” options was run to remove reads matching either to known vectors included in the UniVec collection (http://www.ncbi.nlm.nih.gov/VecScreen/UniVec.html) or to contamination with *E. coli* K12 genome (NC_000913). Reads corresponding to *N. tabacum* mitochondrial genome (NC_006581) or to *N. tabacum* plastid genome (NC_001879), reads shorter than 100 bases, and reads with more than 3% of undetermined bases were also removed. Lower-case masking of simple and complex repeats was performed using Tandem Repeat Finder [52] and RepeatMasker [53], respectively. The library of complex repeats consisted of mips-REdat [16] and repeats from SOL Genomics Network [17]. Sixty bases at each 5’- and 3’-end were trimmed to make sure that they did not contain lower-case masked sequences. Tobacco genome assembly was performed on the set of cleaned and trimmed reads using Celera assembler (http://sourceforge.net/projects/wgs-assembler/?source=directory) with optimized parameters.

Genes were predicted in the genomic contigs with two gene prediction programs: AUGUSTUS [54] and FGENESH [55]. AUGUSTUS was trained on a set of bona fide gene structures of known tobacco genes available in the GenBank. The tobacco-trained version of AUGUSTUS included models for the untranslated regions (5’ and 3’, spliced and unspliced) and the promoter region of a gene. Gene prediction with AUGUSTUS was performed using “hints” based on evidence from tobacco ESTs and cDNAs, homology at the protein level, genomic conservation with four plants (tomato, *Arabidopsis thaliana*, *Medicago trancatula* and rice), and cDNA from the tomato and potato. FGENESH was trained for tobacco by SoftBerry Inc. and run as an *ab initio* gene finder with default parameters. Further iterations of the tobacco genome assembly were also masked with a new *N. tabacum* repeat library constructed *ab initio* by running RepeatScout [56] on the assembled genome.

In order to measure genomic conservation, a 5-way alignment of the genomes of tobacco, tomato, *A. thaliana*, *Medicago truncatula*, and rice was performed using the multiple sequence aligning programs TBA [57] and BLASTZ [58]. A conservation landscape (see Figure 2) was computed using the phylogenetic Hidden Markov Model phastcons [59]. Tobacco ESTs were obtained from several libraries as a part of the TGI.

### Tobacco exon array design

**Figure 10:**
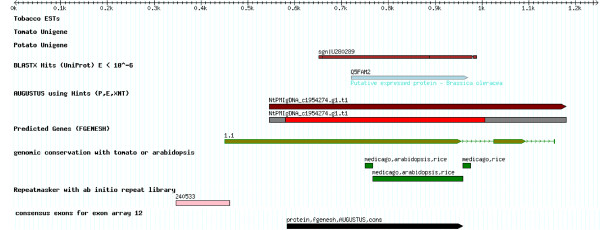
Determination of a consensus exonic region (last track, shown in black) from multiple evidence sources.

The evidence from various sources for exons (transcript sequences, homology, conservation) and against exons (repeats) was integrated towards a set of consensus exonic regions. As not all evidence sources provided exons with exact boundaries and as candidate exonic regions were prioritized by reliability, a custom program was written to combine all evidence sources into a set of non-overlapping exonic regions. For each such exonic region, the supporting evidence was compiled and weighted. A minimum length threshold of 60 bp and a penalty for exonic regions less than 100 bp was applied. An overlap with repeat masked regions was considered negative evidence for an exon. Candidate exonic regions in the genome which that had no other supporting evidence than ESTs were removed. As a consequence, the likely strand could be determined for all exonic regions (Figure 10). FGENESH predictions that were not supported by other evidence and that overlapped a repeat masked region were also removed, and regions that matched transposable elements or other highly repetitive proteins and that overlapped a repeat by more than 25% of their length were filtered out.

The tobacco ESTs were assembled into 55,520 contigs, 8,451 of which were filtered out by the criteria for transposable elements and repeats. An additional 26,882 EST contigs were filtered because they matched a consensus exon candidate from genomic DNA (min 98% identity); in the case of duplicate representation we gave precedence to exon candidates represented in the genomic assembly. In the remaining 20,187 EST contigs we searched for the longest ORF and kept the 16,258 EST contigs with an ORF of minimum length 150. For these, we inferred a putative strand from the ORF.

Exon candidates from genomic DNA and from EST contigs were joined and the genomic candidates were cleaned for redundancies (98% threshold). This resulted in a set of 312,053 exon candidates, 12,925 of which were represented by ESTs, but were not included in the genome assembly.

### Field trials and GGE analysis

Varieties were attributed to subplots using a completely randomized block design. The experimental unit was a pooled sample of 20 plants. The objective of the field trials was to study the variety (genetic make-up) effect on Cd uptake. Forty-five varieties were grown in four environments (two countries, Poland and the Philippines, over two years). In each country, two fields in which burley and flue-cured cultivation practices were used.

Fields were designed by completely randomized block design, with six Blocks and 45 Rows. Plants in each block were sampled at medium stalk position and a pooled sample was created for each block and analyzed by Inductively Coupled Plasma Mass Spectrometry (ICP-MS) for Cd content in leaves. The data were modeled as Cd~E+G+GxE+e, with E being a random effect (G=Genotype, E=Environment=Field x Year). Differences in soil composition and irrigation are known to impact Cd bioavailability creating a confounding with the genotype effect under study, therefore, prior to the GGE analysis [60], the data were corrected by Generalized Additive Models (GAM) [61] for the spatial effect within the field (X~1+Variety+s(Rows,Columns)+e, where s is a cubic regression spline) and then scaled by Environment. Modeling the spatial effect showed its importance in the majority of the fields (see Additional file 2: Figure S1 for an example).

### Plant material and growth (V21 vs. V5 Experiment)

Tobacco seeds of V5 and V21 varieties were germinated in floating trays and grown for three weeks before being transferred to 5 liter pots. Two soils were prepared, one a peaty soil (minus Cd soil, referred to as Soil 1) and the other a soil mixture containing half of the same peaty soil and half of an agricultural clay soil from Poland (pH = 5.1) containing more bioavailable Cd (plus Cd soil, referred to as Soil 2). To ensure that there were no toxic Cd effects and to allow the monitoring of differential Cd accumulation in both tobacco varieties, the soils did not exceed 0.12 mg/kg total Cd. In each soil, six plants were planted and placed on a table according to a randomized pot design. Plants were watered daily on an automatic drip with a full fertilizer (93.03 mg N l^-1^, 49.94 mg P_2_O_5_ l^-1^, 172.27 mg K_2_O l^-1^, 13.67 mg Mg l^-1^, 62.03 mg Ca l^-1^, 27.62 mg S l^-1^ and microelements: 0.472 mg Fe-EDTA l^-1^, 0.309 mg Mn l^-1^, 0.147 mg Zn l^-1^, 0.122 mg B l^-1^, 0.027 mg Cu l^-1^, 0.027 mg Mo l^-1^). The illumination cycle was 14/10 day/night with 24°C during the day and 20°C during the night. After nine weeks of growth and just before flowering, a pool of leaf disks at leaf stalk positions 5, 6 and, 7 and one lateral root were collected simultaneously from each plant. Full leaves 5, 6, and 7 were then collected and dried at 65°C in an oven for three days.

### Preparation of RNA samples

Approximately 100 μg of plant tissue was placed in a 1.5 ml Eppendorf tube (1/3 in volume), and snap frozen in liquid nitrogen. RNA extraction was performed by two methods: Trizol® for microarray analysis (see below) and Qiagen RNeasy Plant mini kit for semi-quantitative RT-PCR.

In case of RNA extraction for microarray experiment, 400 μl of Trizol® reagent (Invitrogen) was added to the frozen plant tissue and the sample was ground to homogeneity before an additional 600 μl of Trizol® was added. Samples were vortexed for 15 s, then incubated at for 5 min room temperature. 200 μl of chloroform was added and the tubes were gently mixed before centrifugation (12,000 × *g*) at 4°C for 15 min. The aqueous phase was transferred to an Eppendorf tube containing 500 μl of isopropyl alcohol. After 15 min incubation on ice, RNA was pelleted by centrifugation at 4°C for 10 min. The RNA pellet was finally washed with 1 ml of 70% ethanol, and centrifuged for five minutes a 7,500 × *g*. After air-drying, RNA was resuspended in 50 μl of nucleotide-free H_2_O. RNA concentration was analyzed by measuring optical density (OD) at 260 nm. RNA quality control was determined by OD260/OD280 and using Agilent 2100 Bioanalyzer before cDNA preparation.

### Microarray hybridization

Frozen samples (−80°C) packed in dry ice were sent to DNA-Vision (Charleroi, Belgium). Total RNA isolation using a Trizol® method (Invitrogen 155596–018), microarray hybridization and quality checked by Agilent 2100 Bioanalyzer were executed at DNA-Vision. Affymetrix hybridizations were performed using Affymetrix kits with catalog numbers 900652 and 900454; probe labeling was checked as suggested by the manufacturer.

### Microarray data analysis

An in-house QC pipeline was developed to assess the quality of the gene-expression data. In addition to the standard quality metrics suggested by Affymetrix http://media.affymetrix.com/support/technical/whitepapers/exon_gene_arrays_qa_whitepaper.pdf, an in-house QC metrics including probe-level models, Normalized Unscaled Standard Error (NUSE) and Relative Log Expression (RLE) plots [62], and the analysis of DABG results http://media.affymetrix.com/support/technical/whitepapers/exon_background_correction_whitepaper.pdf were used. As the exon array design had no mismatch probes, summarization was performed using Robust Multiarray Average (RMA) method [63]. A total of 272,342 probeset expression values were generated, and DABG P-values were computed to assess the significance of the signal obtained for each probeset. This involved the background probes that are spread over the chip. These random probes have a varying GC content http://media.affymetrix.com/support/technical/whitepapers/exon_background_correction_whitepaper.pdf. The QC pipeline involves a combination of Affymetrix Power Tools (APT) http://www.affymetrix.com/partners_programs/programs/developer/tools/powertools.affx and Bioconductor packages, for which the Tobacco Exon Array (TobArray520623F) cdf environment was created. Once the expression values were available, differential gene expression analysis was performed using moderated t-statistics in linear model LIMMA [64].

In addition, gene sets were defined by first annotating probesets by homology to *A. thaliana* genes and using *A. thaliana* gene sets [http://raetschlab.org//suppl/kirmes/a.thaliana-geneset-FASTAs.zip/view.html], and mean-rank gene-set enrichment analysis was performed.

### Cd and Zn chemical analysis

Cd and Zn metal analyses were performed by ALS Group Czech Republic (Prague, http://www.alsglobal.com/). Prior to the metal determination, the samples were homogenized and then digested in organic matrices according to standard operating procedure (SOP) from ALS Group. Cd and Zn concentrations were determined by Inductively Coupled Plasma Optical Emission Spectrometry (ICP-OES) or ICP-MS according to the manufacturer’s instructions.

### Semi-quantitative RT-PCR method

Total RNA was treated with DNAse (RQ1, RNAse free, Promega Catalys, Wallisellen, Switzerland), followed by M-MLV reverse transcriptase (RNAse H minus, point mutant, Promega, Catalys) according to the manufacturer’s recommendation and stored at −20°C. An aliquot of the cDNAs diluted at 1/10 was used in the PCR reaction. After 2 min denaturation at 95°C, 30 PCR cycles (95°C for 30 s, 54°C for 30 s and 72°C for 30 s) were run. The PCR reactions were performed in a final volume of 25 μl containing 1 μM of both forward and reverse primers, 1 U Hot Start Go Taq DNA polymerase 2x Mix (Promega, Wallisellen, Switzerland). The amplified PCR products have a size between 200 and 300 bp. As internal control, tubulin A6 (house-keeping gene) transcripts were amplified using forward 5’- ATTTGTTGACTGGTGCCCAAC and reverse 5’- TCTTCATCGTCAACTTCAGCA primers. The three other transcripts subjected to semi-quantitative RT-PCR were NtPMIa1g222869e1 (gi|48761132) corresponding to a metallophosphatase using the forward 5’-ACAATGCTAGCTTCGGTTATGG and reverse 5’-GGCTCTGCTTCTGTTTTTGTCT primers, NtPMIa1g11112e1_s matching with the protein At4g24110 using the forward 5’-TGTGGCAGCAAATATTTCAAAG and reverse 5’-TCGGATCTTGGAGTCGTTAATC primers and NtPMIa1g68186e1 corresponding to Q9ZS31 (NL27 in *S. tuberosum*) and the *Nicotiana glutinosa* virus resistance (N) gene using the forward 5’-GCAGACTGTATTCGGCATATTG and reverse 5’-TGCTATTGTGGTTTTACCCATTC primers. PCR products were separated by 1.5% agarose gel electrophoresis and visualized by staining with GelRed Nucleic Acid Stain (Chemie Brunswig, Basel, Switzerland).

### Availability of supporting data

The accession number from Gene Expression Omnibus (GEO) GSE42319 (http://www.ncbi.nlm.nih.gov/geo/query/acc.cgi?acc=GSE42319) provides access to all CEL files and meta-data sheet for the experiments described in the manuscript. In addition, GEO platform GPL16290 accession number (http://www.ncbi.nlm.nih.gov/geo/query/acc.cgi?acc=GPL16290) contains all other files (probeset annotations, Affymetrix CDF, and Affymetrix 1lq) necessary to process CEL files generated from Tobacco Exon Array experiments.

## Competing interests

Authors FM, LB, AC, IG, MCP, and NVI are employees of Philip Morris International. Research described in this article was supported by Philip Morris International.

## Authors’ contributions

FM, LB, and NVI conceived the project and wrote the manuscript. FM analyzed the exon array and field data and were established the field designs. LB carried out and supervised greenhouse experiments, material collection, microarray hybridization, and Cd analysis. AC performed RT-PCR analysis of the samples. NVI, MS, and IG performed annotation of the tobacco genome and the exon sequence analysis. NVI and IG communicated with Affymetrix on the chip design. MCP contributed to the project conception and manuscript writing. All authors have read and approved the final manuscript.

## Author’s information

This work is dedicated to the memory of Dr. Nicolas Lugon-Moulin.

## Supplementary Material

Additional file 1: Table S1 Average expression of exons for each variety in leaves. (Variety effect, threshold set to 5 and fdr<0.05). **Table S2.** Average expression of exons for each variety in roots (Variety effect, threshold set to 5 and fdr<0.05). Table S3. Significant gene sets for the variety effect in leaves, roots and the soil effect in roots (fdr<0.01). Click here for file

Additional file 2: Figure S1 Distribution of Cd measurements over the subplots of an experimental tobacco field. The field rows (Y-axis) and the field columns (X-axis) define the field subplots. A) Original Cd values by subplots (min 1.6, Q1 2.5, Med 3 Mean 3.026 Q3 3.4 Max 5.6) (from navy to white to red, low to high). B) Predicted spatial effect by GAM (from slate blue to black to yellow, low to high). Click here for file
