# Supplementary material for: Design of a tobacco exon array with application to investigate the differential cadmium accumulation property in two tobacco varieties
Source: BMC Genomics. 2012 Nov 28;13:674. doi: 10.1186/1471-2164-13-674 (PMC3602038; doi:10.1186/1471-2164-13-674)
Supplement: Additional file 1: Table S1 — Average expression of exons for each variety in leaves. (Variety effect, threshold set to 5 and fdr<0.05). Table S2. Average expression of exons for each variety in roots (Variety effect, threshold set to 5 and fdr<0.05). Table S3. Significant gene sets for the variety effect in leaves, roots and the soil effect in roots (fdr<0.01). [file 1471-2164-13-674-S1.docx]

Additional Table 1. Average expression of exons for each variety in leaves. (Variety effect, threshold set to 5 and fdr<0.05)

| **LEAVES** ID | V5 | V21 | Uniprot_Accession_ID | TAIR_ID | Annotation |
| --- | --- | --- | --- | --- | --- |
| NtPMIa1g117512e2_st | 2.37 | 7.990328333 |  |  |  |
| NtPMIa1g121372e1_st | 1.76 | 6.95 |  |  |  |
| NtPMIa1g126746e1_st | 1.27 | 6.35 | Q8S3E0 | AT3G07340.1 | \| UNIPROT:Putative bHLH transcription factor - Arabidopsis thaliana (Mouse-ear cress) \| TAIR:basic helix-loop-helix (bHLH) family protein |
| NtPMIa1g13156e1_s_st | 3.57 | 9.75 | Q2R211 | AT5G55150.1 | \| UNIPROT:F-box domain containing protein, expressed (Os11g0584100 protein) - Oryza sativa (japonica cultivar-group) \| TAIR:F-box family protein |
| NtPMIa1g13630e1_st | 8.52 | 2.69 | Q9ZV99 | AT1G78810.1 | \| UNIPROT:F9K20.15 - Arabidopsis thaliana (Mouse-ear cress) \| TAIR:unknown protein |
| NtPMIa1g141115e1_st | 6.60 | 1.10 | A1Y9R2_SOLLC |  | \| UNIPROT:Truncated NBS-LRR resistance protein-like protein - Solanum lycopersicum (Tomato) (Lycopersicon esculentum) |
| NtPMIa1g141258e1_st | 8.57 | 3.50 |  |  |  |
| NtPMIa1g142202e1_st | 7.92 | 2.79 | A1Y9R1_SOLLC |  | \| UNIPROT:NBS-LRR resistance protein-like protein - Solanum lycopersicum (Tomato) (Lycopersicon esculentum) |
| NtPMIa1g144143e1_st | 2.95 | 8.23 |  |  |  |
| NtPMIa1g1459e1_s_st | 2.97 | 8.78 | Q7F1L8 |  | \| UNIPROT:Putative membrane related protein CP5 - Oryza sativa (japonica cultivar-group) |
| NtPMIa1g155191e1_x_st | 7.16 | 1.75 |  |  |  |
| NtPMIa1g156424e1_st | 8.10 | 2.02 |  |  |  |
| NtPMIa1g172169e2_st | 4.01 | 9.45 | Q3Y6V1_TOBAC | AT4G23990.1 | \| UNIPROT:Cellulose synthase-like protein CslG - Nicotiana tabacum (Common tobacco) \| TAIR:ATCSLG3, cellulose synthase/ transferase/ transferase, transferring glycosyl groups |
| NtPMIa1g172343e1_st | 3.80 | 8.60 |  | AT4G01100.1 | \| TAIR:ADNT1 (ADENINE NUCLEOTIDE TRANSPORTER 1), ADP transmembrane transporter/ AMP transmembrane transporter/ ATP transmembrane transporter/ binding |
| NtPMIa1g172946e1_st | 3.87 | 9.43 |  |  |  |
| NtPMIa1g174236e1_st | 8.22 | 1.33 |  |  |  |
| NtPMIa1g176060e1_s_st | 5.97 | 1.78 |  |  |  |
| NtPMIa1g177181e2_s_st | 2.35 | 7.99 | Q93ZZ1_ARATH |  | \| UNIPROT:Hypothetical protein At3g23080 - Arabidopsis thaliana (Mouse-ear cress) |
| NtPMIa1g178309e2_st | 2.37 | 10.83 | A0FCS1_9ROSA | AT1G32900.1 | \| UNIPROT:Granule-bound starch synthase (Fragment) - Chamaebatiaria millefolium \| TAIR:starch synthase, putative |
| NtPMIa1g185293e1_st | 7.91 | 2.11 | Q4W6T7_PETHY |  | \| UNIPROT:Polypeptide with an integrase domain - Petunia hybrida (Petunia) |
| NtPMIa1g185616e2_st | 2.65 | 9.92 |  |  |  |
| NtPMIa1g19242e1_st | 1.70 | 7.96 | Q5JZR1 |  | \| UNIPROT:N-rich protein - Glycine max (Soybean) |
| NtPMIa1g200653e1_st | 2.54 | 8.57 | Q1S2S7_MEDTR | AT1G34000.1 | \| UNIPROT:Hypothetical protein - Medicago truncatula (Barrel medic) \| TAIR:OHP2 (ONE-HELIX PROTEIN 2) |
| NtPMIa1g201799e1_st | 10.96 | 5.86 |  |  |  |
| NtPMIa1g202115e1_st | 2.49 | 6.67 | Q9FTV5_ORYSA | AT1G08230.2 | \| UNIPROT:Proline transport protein 2-like (Os01g0621200 protein) - Oryza sativa (japonica cultivar-group) \| TAIR:amino acid transporter family protein |
| NtPMIa1g205560e1_st | 1.67 | 10.63 |  |  |  |
| NtPMIa1g207696e1_st | 7.32 | 1.99 | Q38QB6_SOLBU |  | \| UNIPROT:Late blight resistance protein Rpi-blb2 - Solanum bulbocastanum (Wild potato) |
| NtPMIa1g208723e2_st | 6.22 | 2.54 | Q84UU3_ARATH | AT1G79460.1 | \| UNIPROT:Terpene synthase - Arabidopsis thaliana (Mouse-ear cress) \| TAIR:GA2 (GA REQUIRING 2), ent-kaurene synthase |
| NtPMIa1g213558e1_st | 8.40 | 3.27 | Q7RFY0_PLAYO | AT1G70490.1 | \| UNIPROT:ADP-ribosylation factor - Plasmodium yoelii yoelii \| TAIR:ARFA1D, GTP binding / phospholipase activator/ protein binding |
| NtPMIa1g213558e2_st | 6.33 | 1.63 | Q7RFY0_PLAYO | AT1G70490.1 | \| UNIPROT:ADP-ribosylation factor - Plasmodium yoelii yoelii \| TAIR:ARFA1D, GTP binding / phospholipase activator/ protein binding |
| NtPMIa1g21406e1_s_st | 3.82 | 8.33 | Q1S7E5 |  | \| UNIPROT:MtN3 and saliva related transmembrane protein - Medicago truncatula (Barrel medic) |
| NtPMIa1g218825e1_st | 8.65 | 2.91 |  |  |  |
| NtPMIa1g22326e1_st | 9.33 | 1.39 | Q1RTP6 | AT5G18130.1 | \| UNIPROT:Hypothetical protein - Medicago truncatula (Barrel medic) \| TAIR:unknown protein |
| NtPMIa1g22326e2_st | 9.34 | 3.73 | Q1RTP6 |  | \| UNIPROT:Hypothetical protein - Medicago truncatula (Barrel medic) |
| NtPMIa1g24937e2_st | 2.29 | 7.34 |  |  |  |
| NtPMIa1g25942e2_s_st | 2.52 | 7.24 | Q9XFM3 | AT4G10850.1 | \| UNIPROT:Putative MtN3-like protein - Dianthus caryophyllus (Carnation) (Clove pink) \| TAIR:nodulin MtN3 family protein |
| NtPMIa1g38206e1_st | 8.91 | 1.99 | A1Y9Q6 |  | \| UNIPROT:NBS-LRR resistance protein-like protein - Solanum sp. VFNT |
| NtPMIa1g38691e1_st | 1.57 | 6.49 |  |  |  |
| NtPMIa1g39836e1_st | 8.96 | 3.43 | Q1SB21 | AT3G23790.1 | \| UNIPROT:Hypothetical protein - Medicago truncatula (Barrel medic) \| TAIR:AMP-binding protein, putative |
| NtPMIa1g42181e2_s_st | 8.62 | 2.49 |  |  |  |
| NtPMIa1g52548e2_s_st | 7.32 | 1.66 | Q10HY1 |  | \| UNIPROT:MATE efflux family protein, expressed - Oryza sativa (japonica cultivar-group) |
| NtPMIa1g54862e1_st | 1.59 | 8.40 | Q40392 |  | \| UNIPROT:TMV resistance protein N - Nicotiana glutinosa (Tobacco) |
| NtPMIa1g6169e1_st | 7.83 | 2.17 | Q9M451 |  | \| UNIPROT:Calmodulin-binding protein (Fragment) - Cicer arietinum (Chickpea) (Garbanzo) |
| NtPMIa1g66484e1_st | 2.90 | 8.49 | Q8W237 | AT1G07260.1 | \| UNIPROT:Betanidin 6-O-glucosyltransferase - Dorotheanthus bellidiformis (Livingstone daisy) \| TAIR:UGT71C3 (UDP-GLUCOSYL TRANSFERASE 71C3), UDP-glycosyltransferase/ quercetin 3-O-glucosyltransferase/ transferase, transferring glycosyl groups |
| NtPMIa1g68186e1_st | 2.80 | 9.04 | Q9ZS31 | AT5G17680.1 | \| UNIPROT:NL27 - Solanum tuberosum (Potato) \| TAIR:disease resistance protein (TIR-NBS-LRR class), putative |
| NtPMIa1g68230e2_st | 7.58 | 1.86 | Q8LPF7 | AT1G78800.1 | \| UNIPROT:At1g78800/F9K20_16 - Arabidopsis thaliana (Mouse-ear cress) \| TAIR:glycosyl transferase family 1 protein |
| NtPMIa1g68891e1_st | 6.93 | 1.81 | Q94KA9 | AT1G09270.1 | \| UNIPROT:Importin alpha 2 - Capsicum annuum (Bell pepper) \| TAIR:IMPA-4 (IMPORTIN ALPHA ISOFORM 4), binding / protein transporter |
| NtPMIa1g69413e1_st | 1.52 | 6.39 |  |  |  |
| NtPMIa1g7309e1_st | 7.21 | 1.53 | Q1P9R7 |  | \| UNIPROT:Root-knot nematode resistance protein - Capsicum annuum (Bell pepper) |
| NtPMIa1g74015e1_s_st | 8.52 | 1.44 |  |  |  |
| NtPMIa1g75749e2_st | 1.31 | 7.06 |  |  |  |
| NtPMIa1g76486e1_st | 1.55 | 6.90 |  |  |  |
| NtPMIa1g79251e2_st | 10.75 | 4.03 | O49683 |  | \| UNIPROT:Hypothetical protein AT4g17840 - Arabidopsis thaliana (Mouse-ear cress) |
| NtPMIa1g79251e4_st | 10.84 | 3.96 | O49683 |  | \| UNIPROT:Hypothetical protein AT4g17840 - Arabidopsis thaliana (Mouse-ear cress) |
| NtPMIa1g79251e5_st | 9.83 | 1.44 | O49683 | AT4G17840.1 | \| UNIPROT:Hypothetical protein AT4g17840 - Arabidopsis thaliana (Mouse-ear cress) \| TAIR:unknown protein |
| NtPMIa1g79618e10_st | 8.08 | 3.96 | Q9FRW5 |  | \| UNIPROT:Aspartic proteinase 5 - Nepenthes alata (Winged pitcher plant) |
| NtPMIa1g79618e5_st | 8.31 | 4.41 | Q9FRW5 |  | \| UNIPROT:Aspartic proteinase 5 - Nepenthes alata (Winged pitcher plant) |
| NtPMIa1g79618e9_st | 9.65 | 5.71 | Q9FRW5 | AT4G04460.1 | \| UNIPROT:Aspartic proteinase 5 - Nepenthes alata (Winged pitcher plant) \| TAIR:aspartyl protease family protein |
| NtPMIa1g8207e1_st | 2.50 | 8.13 | Q6L439 | AT1G50180.1 | \| UNIPROT:Putative late blight resistance protein homolog R1A-4 - Solanum demissum (Wild potato) \| TAIR:disease resistance protein (CC-NBS-LRR class), putative |
| NtPMIa1g82578e5_st | 3.12 | 10.30 | Q9MAQ0 | AT1G32900.1 | \| UNIPROT:Probable granule-bound starch synthase 1, chloroplast precursor (EC 2.4.1.242) (Granule-bound starch synthase I) (GBSS-I) - Arabidopsis thaliana (Mouse-ear cress) \| TAIR:starch synthase, putative |
| NtPMIa1g82578e7_st | 3.11 | 8.30 | Q9MAQ0 |  | \| UNIPROT:Probable granule-bound starch synthase 1, chloroplast precursor (EC 2.4.1.242) (Granule-bound starch synthase I) (GBSS-I) - Arabidopsis thaliana (Mouse-ear cress) |
| NtPMIa1g8450e1_st | 1.70 | 7.52 | Q9FK24 | AT4G14500.1 | \| UNIPROT:Membrane related protein-like - Arabidopsis thaliana (Mouse-ear cress) \| TAIR:FUNCTIONS IN: molecular_function unknown, INVOLVED IN: biological_process unknown, LOCATED IN: cellular_component unknown, EXPRESSED IN: 24 plant structures, EXPRESSED DURING: 15 growth stages, CONTAINS InterPro DOMAIN/s: Lipid-binding START (InterPro:IPR002913), BEST Arabidopsis thaliana protein match is: unknown protein (TAIR:AT3G23080.1), Has 257 Blast hits to 256 proteins in 63 species: Archae - 0, Bacteria - 0, Metazoa - 167, Fungi - 0, Plants - 83, Viruses - 0, Other Eukaryotes - 7 (source: NCBI BLink). |
| NtPMIa1g92431e1_st | 7.24 | 2.20 |  |  |  |
| NtPMIa1g93612e1_st | 2.05 | 7.52 | Q9FIV9 |  | \| UNIPROT:TMV resistance protein N - Arabidopsis thaliana (Mouse-ear cress) |
| NtPMIa1g93612e2_st | 1.51 | 7.27 | Q9FIV9 | AT4G33300.1 | \| UNIPROT:TMV resistance protein N - Arabidopsis thaliana (Mouse-ear cress) \| TAIR:ADR1-L1 (ADR1-like 1), ATP binding / protein binding |
| NtPMIa1g96775e1_st | 1.94 | 8.69 | Q6JBD8 | AT5G45260.1 | \| UNIPROT:N-like protein - Nicotiana tabacum (Common tobacco) \| TAIR:RRS1 (RESISTANT TO RALSTONIA SOLANACEARUM 1), DNA binding / transcription factor |
| NtPMIa1g9686e2_st | 1.87 | 8.07 |  |  |  |

Additional Table 2. Average expression of exons for each variety in roots (Variety effect, threshold set to 5 and fdr<0.05)

| ID **ROOT** | V5 | V21 | Uniprot_Accession_ID | TAIR_ID | Annotation |
| --- | --- | --- | --- | --- | --- |
| NtPMIa1g11112e1_s_st | 3.17 | 10.261434 | Q8GXX1 | AT4G24110.1 | \| UNIPROT:Hypothetical protein At4g24110/T19F6_100 - Arabidopsis thaliana (Mouse-ear cress) \| TAIR:unknown protein |
| NtPMIa1g11112e1_st | 1.56 | 10.04 | Q8GXX1 | AT4G24110.1 | \| UNIPROT:Hypothetical protein At4g24110/T19F6_100 - Arabidopsis thaliana (Mouse-ear cress) \| TAIR:unknown protein |
| NtPMIa1g116916e1_st | 6.53 | 2.10 | Q9ZNU5 | AT5G62350.1 | \| UNIPROT:Hypothetical protein At2g01610 - Arabidopsis thaliana (Mouse-ear cress) \| TAIR:invertase/pectin methylesterase inhibitor family protein / DC 1.2 homolog (FL5-2I22) |
| NtPMIa1g117512e2_st | 2.12 | 8.47 |  |  |  |
| NtPMIa1g126734e1_s_st | 2.20 | 7.74 | Q8LMR9 | AT1G01030.1 | \| UNIPROT:Putative RAV-like B3 domain DNA binding protein (Os03g0120900 protein) (B3 DNA binding domain containing protein, expressed) - Oryza sativa (japonica cultivar-group) \| TAIR:NGA3 (NGATHA3), transcription factor |
| NtPMIa1g126746e1_st | 1.29 | 8.23 | Q8S3E0 | AT3G07340.1 | \| UNIPROT:Putative bHLH transcription factor - Arabidopsis thaliana (Mouse-ear cress) \| TAIR:basic helix-loop-helix (bHLH) family protein |
| NtPMIa1g13156e1_s_st | 4.27 | 9.04 | Q2R211 | AT5G55150.1 | \| UNIPROT:F-box domain containing protein, expressed (Os11g0584100 protein) - Oryza sativa (japonica cultivar-group) \| TAIR:F-box family protein |
| NtPMIa1g13630e1_st | 8.92 | 2.71 | Q9ZV99 | AT1G78810.1 | \| UNIPROT:F9K20.15 - Arabidopsis thaliana (Mouse-ear cress) \| TAIR:unknown protein |
| NtPMIa1g137407e2_s_st | 1.11 | 5.92 | Q2MJ20_MEDTR |  | \| UNIPROT:Cytochrome P450 monooxygenase CYP716A12 - Medicago truncatula (Barrel medic) |
| NtPMIa1g1459e1_s_st | 2.68 | 8.65 | Q7F1L8 |  | \| UNIPROT:Putative membrane related protein CP5 - Oryza sativa (japonica cultivar-group) |
| NtPMIa1g155191e1_x_st | 7.56 | 1.74 |  |  |  |
| NtPMIa1g156424e1_st | 7.63 | 1.84 |  |  |  |
| NtPMIa1g157518e1_s_st | 3.18 | 7.95 | Q60FE9_DIACA | AT5G59590.1 | \| UNIPROT:UDP-glucose: chalcononaringenin 2'-O-glucosyltransferase - Dianthus caryophyllus (Carnation) (Clove pink) \| TAIR:UGT76E2 (UDP-GLUCOSYL TRANSFERASE 76E2), UDP-glycosyltransferase/ quercetin 3-O-glucosyltransferase/ quercetin 7-O-glucosyltransferase |
| NtPMIa1g172343e1_st | 2.20 | 7.75 |  | AT4G01100.1 | \| TAIR:ADNT1 (ADENINE NUCLEOTIDE TRANSPORTER 1), ADP transmembrane transporter/ AMP transmembrane transporter/ ATP transmembrane transporter/ binding |
| NtPMIa1g178309e2_st | 2.45 | 8.25 | A0FCS1_9ROSA | AT1G32900.1 | \| UNIPROT:Granule-bound starch synthase (Fragment) - Chamaebatiaria millefolium \| TAIR:starch synthase, putative |
| NtPMIa1g185177e1_st | 3.13 | 9.10 | O82607_ARATH |  | \| UNIPROT:T2L5.9 protein - Arabidopsis thaliana (Mouse-ear cress) |
| NtPMIa1g185616e2_st | 2.73 | 9.49 |  |  |  |
| NtPMIa1g19242e1_st | 1.52 | 7.93 | Q5JZR1 |  | \| UNIPROT:N-rich protein - Glycine max (Soybean) |
| NtPMIa1g195491e1_st | 2.23 | 8.08 |  |  |  |
| NtPMIa1g205560e1_st | 1.58 | 8.10 |  |  |  |
| NtPMIa1g209835e1_st | 2.26 | 7.42 | O22993_ARATH | AT4G23940.1 | \| UNIPROT:Cell division protein isolog (Cell division protein-like) - Arabidopsis thaliana (Mouse-ear cress) \| TAIR:FtsH protease, putative |
| NtPMIa1g211470e1_s_st | 2.67 | 7.78 | Q5Z4V2_ORYSA | AT1G36730.1 | \| UNIPROT:Putative eukaryotic translation initiation factor 5 - Oryza sativa (japonica cultivar-group) \| TAIR:eukaryotic translation initiation factor 5, putative / eIF-5, putative |
| NtPMIa1g213558e1_st | 10.16 | 3.05 | Q7RFY0_PLAYO | AT1G70490.1 | \| UNIPROT:ADP-ribosylation factor - Plasmodium yoelii yoelii \| TAIR:ARFA1D, GTP binding / phospholipase activator/ protein binding |
| NtPMIa1g213558e2_st | 8.19 | 2.39 | Q7RFY0_PLAYO | AT1G70490.1 | \| UNIPROT:ADP-ribosylation factor - Plasmodium yoelii yoelii \| TAIR:ARFA1D, GTP binding / phospholipase activator/ protein binding |
| NtPMIa1g23471e1_st | 7.97 | 1.44 |  |  |  |
| NtPMIa1g24937e2_st | 1.96 | 7.30 |  |  |  |
| NtPMIa1g25957e1_st | 1.82 | 7.05 |  |  |  |
| NtPMIa1g25981e1_st | 2.81 | 9.78 | Q9M051 | AT4G15260.1 | \| UNIPROT:Glucuronosyl transferase-like protein - Arabidopsis thaliana (Mouse-ear cress) \| TAIR:UDP-glucoronosyl/UDP-glucosyl transferase family protein |
| NtPMIa1g39836e1_st | 8.20 | 3.01 | Q1SB21 | AT3G23790.1 | \| UNIPROT:Hypothetical protein - Medicago truncatula (Barrel medic) \| TAIR:AMP-binding protein, putative |
| NtPMIa1g47456e3_st | 1.89 | 7.82 | Q9T012 | AT4G11080.1 | \| UNIPROT:98b like protein - Arabidopsis thaliana (Mouse-ear cress) \| TAIR:high mobility group (HMG1/2) family protein |
| NtPMIa1g48722e2_st | 3.67 | 7.93 | Q94JV8 | AT5G35732.1 | \| UNIPROT:AT2g04793 (At2g04793/At2g04793) (Expressed protein) - Arabidopsis thaliana (Mouse-ear cress) \| TAIR:unknown protein |
| NtPMIa1g55038e3_st | 2.36 | 7.58 | Q9ZWK4 |  | \| UNIPROT:Alcohol dehydrogenase (Fragment) - Brassica oleracea (Wild cabbage) |
| NtPMIa1g60107e2_st | 1.75 | 7.07 | Q1PFR9 | AT1G28040.1 | \| UNIPROT:Zinc finger family protein - Arabidopsis thaliana (Mouse-ear cress) \| TAIR:protein binding / zinc ion binding |
| NtPMIa1g61192e1_st | 5.05 | 10.42 | Q40575 |  | \| UNIPROT:SAR8.2e protein - Nicotiana tabacum (Common tobacco) |
| NtPMIa1g67752e1_st | 7.75 | 2.11 |  |  |  |
| NtPMIa1g68230e2_st | 7.46 | 1.63 | Q8LPF7 | AT1G78800.1 | \| UNIPROT:At1g78800/F9K20_16 - Arabidopsis thaliana (Mouse-ear cress) \| TAIR:glycosyl transferase family 1 protein |
| NtPMIa1g6971e1_st | 1.56 | 6.21 |  |  |  |
| NtPMIa1g74026e1_st | 2.59 | 7.80 | Q0DYN3 | AT1G63970.1 | \| UNIPROT:Os02g0680600 protein - Oryza sativa (japonica cultivar-group) \| TAIR:ISPF, 2-C-methyl-D-erythritol 2,4-cyclodiphosphate synthase |
| NtPMIa1g75749e2_st | 1.49 | 7.44 |  |  |  |
| NtPMIa1g77252e5_s_st | 1.83 | 8.74 | O04614 |  | \| UNIPROT:A_IG002N01.10 protein - Arabidopsis thaliana (Mouse-ear cress) |
| NtPMIa1g79251e1_st | 8.87 | 3.08 | O49683 |  | \| UNIPROT:Hypothetical protein AT4g17840 - Arabidopsis thaliana (Mouse-ear cress) |
| NtPMIa1g79251e2_st | 7.46 | 1.56 | O49683 |  | \| UNIPROT:Hypothetical protein AT4g17840 - Arabidopsis thaliana (Mouse-ear cress) |
| NtPMIa1g8207e1_st | 2.87 | 8.72 | Q6L439 | AT1G50180.1 | \| UNIPROT:Putative late blight resistance protein homolog R1A-4 - Solanum demissum (Wild potato) \| TAIR:disease resistance protein (CC-NBS-LRR class), putative |
| NtPMIa1g83250e5_st | 8.08 | 2.65 | Q2V9B4 | AT4G37070.2 | \| UNIPROT:Patatin-like protein 1 - Solanum tuberosum (Potato) \| TAIR:patatin, putative |
| NtPMIa1g83250e6_st | 6.64 | 1.46 | Q2V9B4 | AT4G37050.1 | \| UNIPROT:Patatin-like protein 1 - Solanum tuberosum (Potato) \| TAIR:PLP4 (PATATIN-LIKE PROTEIN 4), nutrient reservoir |
| NtPMIa1g8450e1_st | 1.69 | 7.30 | Q9FK24 | AT4G14500.1 | \| UNIPROT:Membrane related protein-like - Arabidopsis thaliana (Mouse-ear cress) \| TAIR:FUNCTIONS IN: molecular_function unknown, INVOLVED IN: biological_process unknown, LOCATED IN: cellular_component unknown, EXPRESSED IN: 24 plant structures, EXPRESSED DURING: 15 growth stages, CONTAINS InterPro DOMAIN/s: Lipid-binding START (InterPro:IPR002913), BEST Arabidopsis thaliana protein match is: unknown protein (TAIR:AT3G23080.1), Has 257 Blast hits to 256 proteins in 63 species: Archae - 0, Bacteria - 0, Metazoa - 167, Fungi - 0, Plants - 83, Viruses - 0, Other Eukaryotes - 7 (source: NCBI BLink). |
| NtPMIa1g96775e1_st | 2.01 | 7.05 | Q6JBD8 | AT5G45260.1 | \| UNIPROT:N-like protein - Nicotiana tabacum (Common tobacco) \| TAIR:RRS1 (RESISTANT TO RALSTONIA SOLANACEARUM 1), DNA binding / transcription factor |

Additional Table 3. Significant genesets for the variety effect in leaves, roots and the soil effect in roots (fdr<0.01)

| Leaves Variety effect | Roots Variety effect | Roots soil effect |
| --- | --- | --- |
| ABC transporter | nucleus_snoR | ABC transporter |
| auxin transporter | protein degradation_F-box FBD | AP2/EREBP |
| fatty acid | silencing_miRNA | auxin transporter |
| hormone | sugar_lactose degradation | bHLH |
| light | TF_bHLH | biosynthesis |
| lipid |  | bZIP |
| pathogen |  | cell wall |
| sugar |  | cytoskeleton |
| WRKY |  | domain |
| AMP_AMP |  | GARP |
| cytoskeleton_general |  | HB |
| enzyme_dehydrogenase |  | hormone |
| enzyme_inositol |  | metal |
| enzyme_P450 |  | oxygen |
| enzyme_sorbitol fermentation |  | pathogen |
| hormone_auxin |  | peptide transporter |
| light_chlorophyll |  | RLK/Pelle |
| lipid_general |  | RLK/Pelle LRR |
| nucleus_snoR |  | ST RCLK |
| pathogen_disease |  | stress |
| silencing_miRNA |  | sugar |
| ST_SnRK2 subfamily |  | sugar transporter |
| sugar_lactose degradation |  | UDP |
| sugar_starch |  | WRKY |
| TF_AP2/EREBP B |  | biosynthesis_general |
| TF_AUX/IAA |  | cell wall_ARABINOGALACTAN |
| TF_C2C2-co-like 1 |  | cell wall_chitinase |
| TF_HB |  | cell wall_general |
| TF_WRKY 2 |  | chromatin_GCN5 |
| TR_(1.A.8) Major Intrinsic Protein |  | cytoskeleton_kinesin |
| TR_(2.A.17) Proton-dependent Oligopeptide Transporter |  | cytoskeleton_microtubule |
| TR_(2.A.18) Amino Acid/Auxin Permease |  | cytoskeleton_tubulin |
| TR_(2.A.19) Ca2+:Cation Antiporter |  | degradation_threonine degradation |
| TR_(3.A.1.205) Pleiotropic Drug Resistance protein |  | domain_DUF |
|  |  | domain_FAD (oxidoreductase?) |
|  |  | domain_germin |
|  |  | domain_proline-rich |
|  |  | enzyme_alcohol dehydrogenase |
|  |  | enzyme_ascorbate glutathione cycle |
|  |  | enzyme_dehydrogenase |
|  |  | enzyme_dioxygenase |
|  |  | enzyme_general |
|  |  | enzyme_hydrolase |
|  |  | enzyme_invertase |
|  |  | enzyme_lipase |
|  |  | enzyme_lipases pathway |
|  |  | enzyme_lipoxygenase pathway |
|  |  | enzyme_oxidoreductase |
|  |  | enzyme_P450 |
|  |  | enzyme_sorbitol fermentation |
|  |  | GTPase_rho |
|  |  | hormone_ET |
|  |  | hormone_GA |
|  |  | hormone_general |
|  |  | metal_copper |
|  |  | no system name_no system name |
|  |  | nucleus_snoR |
|  |  | oxygen_oxygenase |
|  |  | pathogen_disease |
|  |  | pathogen_general |
|  |  | pathogen_harpin (disease) |
|  |  | pathogen_MLP (pathogen defense) |
|  |  | pathogen_pathogenesis |
|  |  | peroxidase_peroxidase |
|  |  | plasmodesma_general |
|  |  | protein degradation_F-box |
|  |  | protein degradation_F-box FBD |
|  |  | protein degradation_F-box Kelch repeats |
|  |  | protein degradation_protease |
|  |  | protein degradation_proteinase |
|  |  | protein degradation_subtilase proteolyse |
|  |  | protein interaction_DC1 (protein-protein interaction zinc binding) |
|  |  | putative_NLI interacting |
|  |  | signaling_phospholipase |
|  |  | silencing_miRNA |
|  |  | ST_general |
|  |  | ST_LRR receptor kinase |
|  |  | ST_Raf subfamily |
|  |  | ST_RLK/Pelle DUF26 |
|  |  | ST_RLK/Pelle L-LEC subfamily |
|  |  | ST_RLK/Pelle LRR subfamily |
|  |  | ST_RLK/Pelle LRR-II subfamily |
|  |  | ST_RLK/Pelle LRR-III subfamily |
|  |  | ST_RLK/Pelle LRR-VII subfamily |
|  |  | ST_RLK/Pelle LRR-VIII subfamily |
|  |  | ST_RLK/Pelle LRR-XI subfamily |
|  |  | ST_RLK/Pelle SD |
|  |  | stress_GST |
|  |  | stress_wound |
|  |  | sugar_cellulose biosynthesis |
|  |  | sugar_galactose |
|  |  | sugar_general |
|  |  | sugar_starch |
|  |  | sugar_trehalose biosynthesis |
|  |  | sugar_UDP-glucose conversion |
|  |  | TF_AP2/EREBP B |
|  |  | TF_ARF |
|  |  | TF_bHLH 3 |
|  |  | TF_bZIP 1 |
|  |  | TF_C2H2 C1 |
|  |  | TF_GARP-G2-like |
|  |  | TF_HB |
|  |  | TF_JUMONJI |
|  |  | TF_NAC 4 |
|  |  | TF_WRKY 2 |
|  |  | TR_(0.0.0) unclassified membrane proteins |
|  |  | TR_(1.A.1) Voltage-gated Ion Channel |
|  |  | TR_(1.A.20) gp91phox Phagocyte NADPH Oxidase-associated Cytochrome b558 H+-channel |
|  |  | TR_(1.A.8) Major Intrinsic Protein |
|  |  | TR_(2.A.1.1) Sugar Porter |
|  |  | TR_(2.A.1.9) Phosphate: H+ Symporter |
|  |  | TR_(2.A.17) Proton-dependent Oligopeptide Transporter |
|  |  | TR_(2.A.18) Amino Acid/Auxin Permease |
|  |  | TR_(2.A.19) Ca2+:Cation Antiporter |
|  |  | TR_(2.A.53) Sulfate Permease |
|  |  | TR_(2.A.66) Multidrug/Oligosaccharidyl-lipid/Polysaccharide Flippase |
|  |  | TR_(2.A.7.9) Triose-phosphate Transporter |
|  |  | TR_(3.A.1.201) Multidrug Resistance Exporter (MDR) |
|  |  | TR_(3.A.1.205) Pleiotropic Drug Resistance protein |
|  |  | TR_(3.A.3) P-type ATPase |
|  |  | TR_general |
|  |  | TR_Porter |
|  |  | TR_Transporter |
|  |  | transcription_DUF246 domain containing |
|  |  | transferase_aminotransferase |
|  |  | transferase_Galacturonosyltransferase |
|  |  | transferase_glycosyltransferase |
|  |  | transferase_methlytransferase S-adenosyl |
|  |  | transferase_transferase |
|  |  | transferase_transferase glycosyl |
|  |  | UDP_UDP |
