# Supplementary figures and images for: Design of a tobacco exon array with application to investigate the differential cadmium accumulation property in two tobacco varieties
Source: BMC Genomics. 2012 Nov 28;13:674. doi: 10.1186/1471-2164-13-674 (PMC3602038; doi:10.1186/1471-2164-13-674)

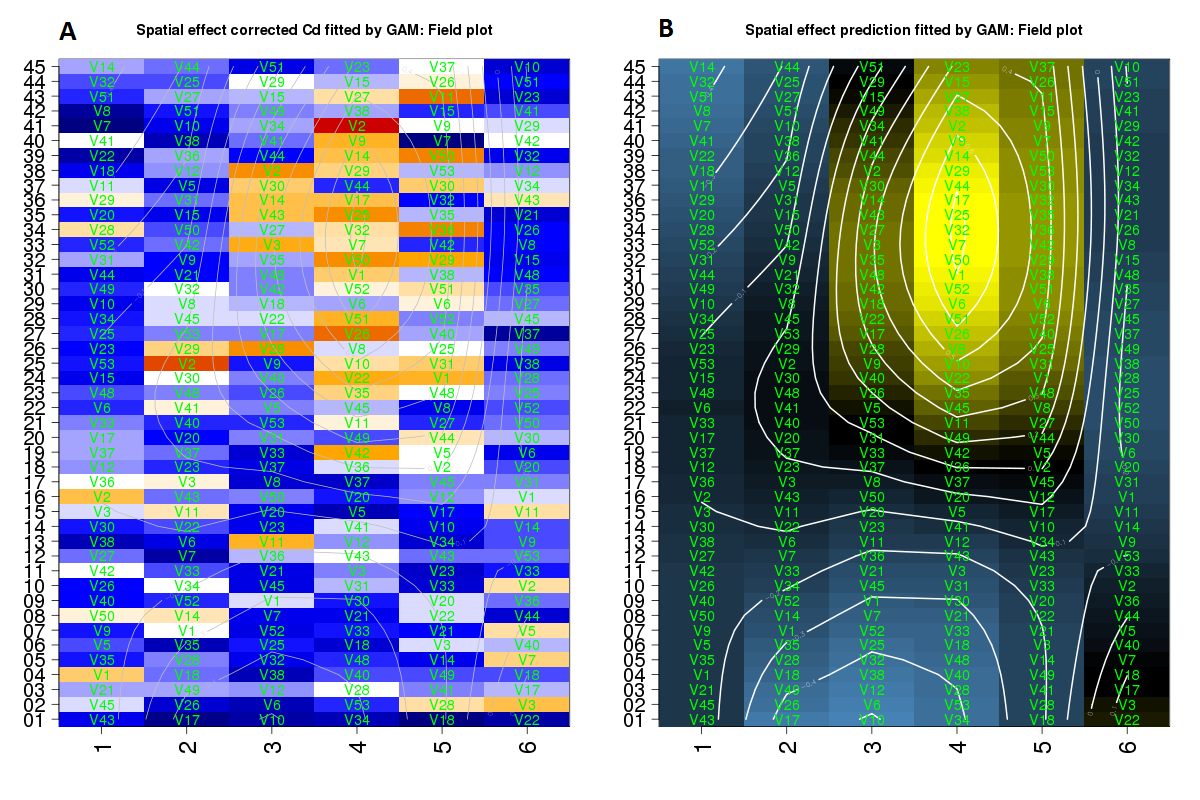

Supplement: Additional file 2: Figure S1 — Distribution of Cd measurements over the subplots of an experimental tobacco field. The field rows (Y-axis) and the field columns (X-axis) define the field subplots. A) Original Cd values by subplots (min 1.6, Q1 2.5, Med 3 Mean 3.026 Q3 3.4 Max 5.6) (from navy to white to red, low to high). B) Predicted spatial effect by GAM (from slate blue to black to yellow, low to high). [file 1471-2164-13-674-S2.png]
